# Email recruitment for chronic pain clinical trials: results from the LAMP trial

**DOI:** 10.1186/s13063-024-08301-8

**Published:** 2024-07-19

**Authors:** John E. Ferguson, Emily Hagel Campbell, Ann Bangerter, Lee J. S. Cross, Kelli D. Allen, Kimberly Behrens, Mariah Branson, Collin Calvert, Jessica K. Friedman, Sierra Hennessy, Laura A. Meis, Brent C. Taylor, Diana J. Burgess

**Affiliations:** 1 Divisions of PM&R and Rehabilitation Science, Department of Rehabilitation Medicine, University of Minnesota, Minneapolis, MN USA; 2 Center for Care Delivery and Outcomes Research, Minneapolis VA Health Care System, Minneapolis, MN 55417 USA; 3 Center of Innovation to Accelerate Discovery and Practice Transformation, Durham VA Health Care System, Durham, NC USA; 4 University of Minnesota Medical School, Minneapolis, MN USA; 5 Thurston Arthritis Research Center, School of Medicine, University of North Carolina at Chapel Hill, Chapel Hill, NC USA; 6 Center for the Study of Healthcare Innovation, Implementation and Policy, VA Greater Los Angeles Health Care System, Los Angeles, CA USA; 7 National Center for Post Traumatic Stress Disorder Women’s Health Sciences Division, Veterans Affairs, MA Boston, USA

**Keywords:** Pragmatic clinical trial, Recruitment, Email, Mindfulness, Chronic pain

## Abstract

**Background:**

Recruitment for clinical trials and large-scale studies is challenging, especially for patients with complex conditions like chronic pain. Email recruitment has the potential to increase efficiency, to reduce costs, and to improve access for underrepresented patient populations. The objective of this study was to examine the effectiveness, efficiency, and equitability of email versus postal mail recruitment for the Learning to Apply Mindfulness to Pain (LAMP) study, a three-site clinical trial of mindfulness-based interventions for chronic pain.

**Methods:**

Patients with chronic pain diagnoses were recruited from three United States Department of Veterans Affairs (VA) facilities using the VA electronic health record (EHR). Recruitment materials were sent using either postal mail (*n* = 7986) or email (*n* = 19,333). Patients in the email recruitment group were also mailed introductory postcards before any emails. Mailing addresses and email addresses were obtained from the EHR. Effectiveness was measured by the response rate of patients who logged into the secure LAMP study website. Efficiency was measured by the number of days from when the recruitment materials were sent to when patients logged into the LAMP portal as well as the estimated costs of each recruitment approach. To assess equitability, we examined whether email recruitment was less effective for underrepresented populations, based on demographic information from the EHR.

**Results:**

Effectiveness—unadjusted response rates were greater for email versus postal-mail recruitment (18.9% versus 6.3%), and adjusted response rates were over three times greater for email recruitment (RR = 3.5, 95% CI 3.1–3.8) based on a multivariable analysis controlling for age, gender, race, ethnicity, rurality, and site. Efficiency—email recruitment had a significantly lower mean response time (1 day versus 8 days) and a lower cost. Equity—email recruitment led to higher response rates for all subpopulations, including older, non-White, Hispanic, rural, and female Veterans.

**Conclusions:**

Email recruitment is an effective, efficient, and equitable way to recruit VA patients to large-scale, chronic pain clinical trials.

**Trial registration:**

Clinical Trial Registration Number: NCT04526158. Patient enrollment began on December 4, 2020.

## Background

Clinical trial recruitment is an active area of study due to its importance in contributing to the success of clinical trials as well as its many practical challenges [1]. Clinical trials with ineffective recruitment efforts can lead to underpowered or failed studies [2] and can have significant financial and ethical implications [3]. Clinical trials often have difficulty recruiting underrepresented patient groups, resulting in study populations that do not reflect the targeted populations [4–6]. Chronic pain clinical trials, in particular, often have difficulty recruiting sufficient sample sizes and recruiting underrepresented patient groups, yet very few studies have investigated the success of different recruitment methods for chronic pain clinical trials [7].

In recent years, digital approaches to clinical trial recruitment (e.g., email, text messages, websites, and social media) have been compared to more traditional approaches (e.g., mailing, phone calls, newspaper advertisements, and media campaigns). The results in terms of response rates, costs, time to recruit participants, and access have been mixed, depending on the specific details of how the digital recruitment tools were implemented [8, 9]. However, several studies have shown that combining digital and traditional recruitment tools may have the potential to improve recruitment outcomes [10, 11].

The Learning to Apply Mindfulness to Pain (LAMP) study is a three-site clinical trial to test the effectiveness of mindfulness-based interventions (MBIs) for chronic pain. Patients with moderate to severe chronic pain were recruited from three U.S. Veterans Affairs facilities and were randomly assigned to two intervention groups (Group MBI and Self-paced MBI), which were compared against usual care. The primary outcome was change in the Brief Pain Inventory (BPI) interference score at 10 weeks, 6 months, and 12 months. Secondary outcomes include changes in pain intensity, global improvement in pain, anxiety, depression, fatigue, post-traumatic stress disorder, physical function, sleep disturbance, and participation in social roles and activities. Additional details can be found in the study protocol paper [12].

Partway through the LAMP study, we switched from traditional postal recruitment to email recruitment, which allowed us to compare the two recruitment modalities in terms of equity, efficiency, and effectiveness in a United States Department of Veterans Affairs (VA) population.

## Methods

Patients were recruited from the Minneapolis VA Health Care System (MVAHCS), Durham VA Health Care System (DVAHCS), and VA Greater Los Angeles Healthcare System (VAGLAHS) if their electronic health record (EHR) showed qualifying pain diagnoses on at least two occasions within the same pain category, at least 90 days part, during the previous two years [12]. The qualifying pain categories were defined using the *International Classification of Diseases, Tenth Revision, Clinical Modification* (ICD-10-CM) diagnostic codes for common pain conditions [13]. To ensure generalizability of the pragmatic clinical trial, minimal exclusion criteria were used. This study is part of the LAMP trial, which was approved by the VA Central Institutional Review Board.

Recruitment materials were sent to six separate waves of patients, recruited at different times between 2020 and 2022. Each wave was a random sample of potentially eligible men and women from the included recruitment sites at that time. Part of the recruitment occurred during the COVID-19 pandemic. The recruitment method started as postal recruitment only for the first two waves, but we decided to try email recruitment for the remaining waves due to an increase in printing and mailing costs. We also felt that email recruitment might integrate better with the technology focus of the study. Women were oversampled from the identified population to try to get approximately even numbers of men and women randomized into the trial. Patients were either sent recruitment materials by postal mail or email, depending on their wave of recruitment. Their postal and email addresses were obtained from the VA EHR. The number of recruitment packets sent for each wave was based on an estimated response rate that would efficiently fill the intervention session times. We also made sure that the group sizes for each wave would not exceed the capacity of the Group MBI intervention facilitators.

Patients in the postal recruitment group, waves 1 and 2, were mailed a packet of documents that included information about the study and instructions for accessing the study website. The mailed packet included an optional quick response (QR) code that could be used to simplify the process of accessing the study website. They were also given contact information and a prefilled postcard to opt out of the study if they wanted. Patients who logged into the study website using a study-specific identifier were prompted to complete the study screener. The patients in the postal recruitment group were from the MVAHCS site and not the other sites.

Patients in the email recruitment group, waves 3–6, were first mailed an introductory postcard, a requirement of our Institutional Review Board (IRB). The postcard notified patients that they would receive an email about participation in the study. They were also given contact information and a website link to opt out of the study if they wanted. Approximately a week after they were sent a postcard, they were sent an email that contained the same information as the packet of documents sent to the postal recruitment group. No one who was sent email recruitment materials requested paper documents. Waves 3 and 4 were patients from the MVAHCS and DVAHCS sites. Waves 5 and 6 were mainly patients from the DVAHCS and VAGLAHS sites.

Reminder postcards were mailed to non-responders in waves 1–3, and reminder emails were sent to non-responders in waves 3, 5, and 6. Reminder emails were not sent to patients in wave 4 because the maximum number of participants who could be included for that wave had already been reached. In line with the pragmatic nature of the study, the reminder methods changed over the different waves as we tried to improve recruitment strategies.

Effectiveness was measured by the response rate of patients to the recruitment materials, where a response was defined as a patient logging into the study website using their study-specific identifier. No more than a single response per patient was recorded in the dataset. Logging into the study website was chosen as a response, as opposed to completing the study screener, because patients may exit the screener early for reasons that do not reflect their engagement with the recruitment method, such as inclusion and exclusion criteria. We then calculated a ratio of email to postal response rates by dividing the email response rate by the postal response rate.

Efficiency was measured by the response time for patients to respond to the recruitment materials as well as the difference in estimated costs. Response time was calculated as the number of days from mailing the packet of study documents to logging into the study website for the postal-recruited group and as the number of days from sending the email for the email-recruited group to logging into the study website. The postcard mailing date was not included in this calculation because patients were unable to log into the study website until they received either the mailed packet or email. To compare response times between email and postal recruitment, we generated box and whisker plots by recruitment strategy. The cost for postal recruitment materials included printing and mailing ten different items in the mailed recruitment packet. The cost for email recruitment materials was primarily the cost of the postcards.

Equity was based on an analysis of response rates by recruitment method across key demographic groups. We coded age, gender, race, ethnicity, and rurality based on the patient’s entry in the VA EHR. VA rurality data is based on the Rural–Urban Commuting Areas (RUCA) system, which classifies United States census tracts using measures of urbanization, population density, and daily commuting. We also conducted a multivariable analysis of response rates controlling for age, gender, race, ethnicity, rurality, and site.

## Results

**Fig. 1:**
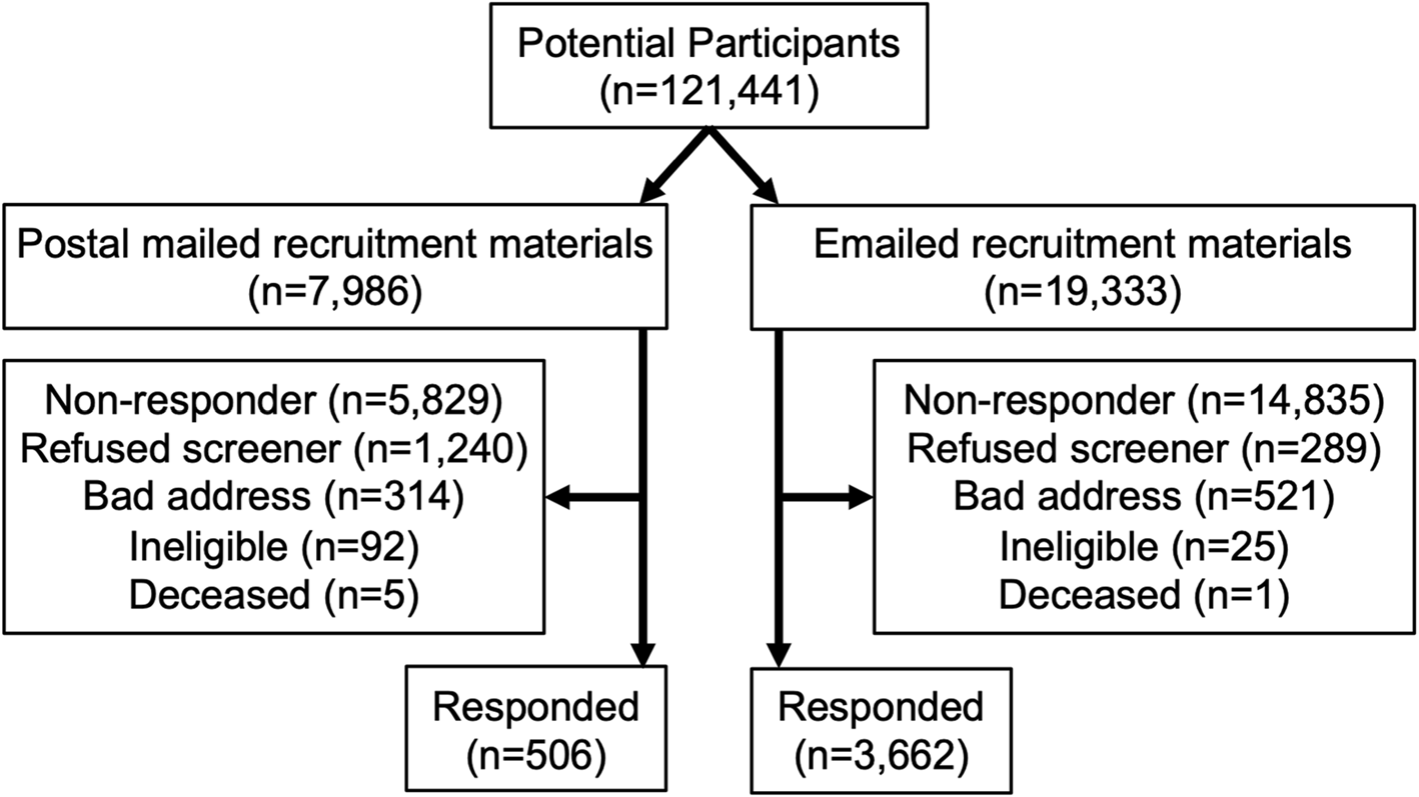
Recruitment flow diagram

We identified 121,441 potential participants from the VA EHR and sent postal mail recruitment materials to 7986 patients and email recruitment materials to 19,333 patients (Fig. 1). Table 1 shows the demographic information for the patients sent recruitment materials. Due to the demographics of the Veterans at the different recruitment sites used for the different waves, the patients sent email materials were younger, more female, more ethnically and racially diverse, and less rural.

**Table 1:** Baseline characteristics of patients sent recruitment materials

	All patients sent recruitment materials (*n* = 27,319)	Patients sent postal materials (*n* = 7986)	Patients sent email materials (*n* = 19,333)
**Age—mean years (SD)**	56.9 (16.2)	62.8 (15.6)	54.5 (15.7)
**Age, *n* (%)**			
< 40	5287 (19.4%)	957 (12.0%)	4330 (22.4%)
40–64	11,814 (43.3%)	2666 (33.4%)	9148 (47.3%)
65 +	10,214 (37.4%)	4363 (54.6%)	5851 (30.3%)
**Gender—female, *n* (%)**	7556 (27.7%)	722 (9.0%)	6834 (35.4%)
**Race, *n* (%)**			
American Indian	259 (1.0%)	84 (1.1%)	175 (0.9%)
Asian	469 (1.7%)	60 (0.8%)	409 (2.1%)
Black	5882 (21.5%)	426 (5.3%)	5456 (28.2%)
Hawaiian	335 (1.2%)	69 (0.9%)	266 (1.4%)
White	18,048 (66.1%)	6753 (84.6%)	11,295 (58.4%)
Multi-race	250 (0.9%)	40 (0.5%)	210 (1.1%)
Unknown	2076 (7.6%)	554 (6.9%)	1522 (7.9%)
**Ethnicity, *n* (%)**			
Hispanic	1670 (6.1%)	138 (1.7%)	1532 (7.9%)
Non-Hispanic	23,632 (86.5%)	7471 (93.6%)	16,161 (83.6%)
Unknown	2017 (7.4%)	377 (4.7%)	1640 (8.5%)
**Rurality, *n* (%)**			
Rural	8878 (32.5%)	3129 (39.2%)	5749 (29.7)
Urban	18,364 (67.2%)	4840 (60.6%)	13,524 (70.0%)
Unknown	77 (0.3%)	17 (0.2%)	60 (0.3%)
**Site, *n* (%)**			
DVAHCS	8093 (29.6%)	0 (0%)	8093 (41.9%)
MVAHCS	13,463 (49.3%)	7986 (100%)	5477 (28.3%)
VAGLAHS	5763 (21.1%)	0 (0%)	5763 (29.8%)

*Abbreviations*: *DVAHCS* Durham VA Health Care System, *MVAHCS* Minneapolis VA Health Care System, *VAGLAHS* VA Greater Los Angeles Healthcare System

### Effectiveness

Unadjusted response rates were higher for email recruitment (18.9%) compared to postal mail recruitment (6.3%). Additionally, in a multivariable analysis controlling for age, gender, race, ethnicity, rurality, and site, the adjusted response rates were over three times greater for email recruitment (RR = 3.5, 95% CI 3.1–3.8).

Most non-responders did not contact the study team. However, 1240 (15.5%) patients in the postal group actively refused the screener, mostly using opt-out postcards, compared with 289 (1.2%) of patients in the email group. Recruitment materials were returned to the study team due to bad address for 314 (3.9%) patients in the postal group and 521 (2.7%) patients in the email group. A small number of patients in both groups or their family members contacted the study team to inform us that the patient was ineligible or deceased. Additionally, a few patients were determined to be ineligible or to have deceased based on a chart review performed by the study team approximately six weeks after the recruitment materials were sent.

Following initial recruitment, 1524 of the 3662 (42%) responders in the email group and 213 of the 506 (42%) responders in the postal group completed the baseline survey, showing that recruitment method did not negatively affect engagement in other study activities. We ultimately randomized 667 (18%) responders from the email group and 144 (28%) responders from the postal mail group into the trial. Due to the unexpectedly high effectiveness of email recruiting, the maximum capacity of the intervention sessions was reached in later waves, and some eligible patients were not randomized to an intervention group in that wave but were included in next recruitment wave.

### Efficiency

**Fig. 2:**
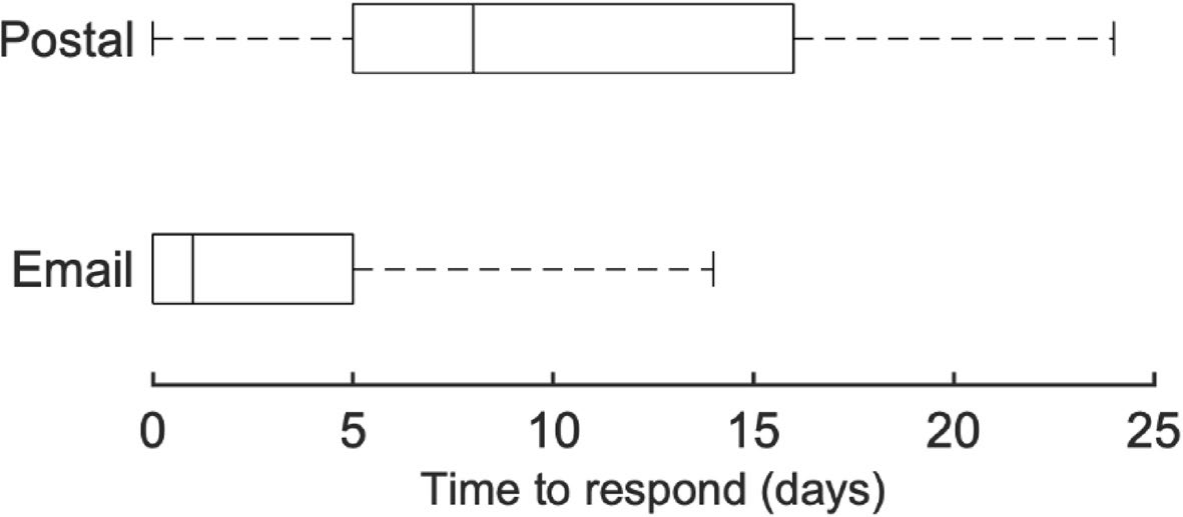
Response time following email or postal mailed recruitment materials. The median is indicated by the vertical line, the interquartile range by the box, and the 2.5th and 97.5th percentiles by the whiskers

The time to respond to the recruitment materials was much shorter for email than postal mail recruitment (Fig. 2). The median time to respond was 1 day for the email group compared to 8 days for the postal group. Many people in the email group responded the same day that the email recruitment materials were sent. The cost of printing and mailing the recruitment materials to the 19,333 patients in the email group would have cost approximately $2.33 per participant, corresponding to a total of approximately $45,000 saved. Additionally, the personnel time required to prepare and send the recruitment materials was estimated to have taken 130–200 h for the 7986 people in the postal group and 5–20 h for the 19,333 people in the email group.

### Equity

Email recruitment had a higher unadjusted response rate than postal mail recruitment for every age, gender, race, ethnicity, and rurality category (Table 2). The unadjusted ratio of email to postal response rates was at least 2.2 for each subpopulation, and the highest unadjusted ratio was 7.1 for Black patients.

**Table 2:** Response rates

	**Postal response rate**	**Email response rate**	**Ratio of email to postal response rates**
**Age**			
< 40	3.9% (37/957)	15.4% (665/4330)	4.0
40–64	7.8% (207/2666)	21.0% (1923/9148)	2.7
65 +	6.0% (262/4363)	18.3% (1071/5851)	3.0
**Gender**			
Female	9.7% (70/722)	21.1% (1443/6834)	2.2
Male	6.0% (436/7264)	17.8% (2219/12499)	3.0
**Race**			
American Indian	6.0% (5/84)	14.9% (26/175)	2.5
Asian	3.3% (2/60)	13.7% (56/409)	4.1
Black	2.3% (10/426)	16.8% (915/5456)	7.1
Hawaiian	4.3% (3/69)	16.9% (45/266)	3.9
White	6.8% (459/6753)	20.8% (2348/11295)	3.1
Multi-race	2.5% (1/40)	15.2% (32/210)	6.1
**Ethnicity**			
Hispanic	5.1% (7/138)	14.4% (220/1532)	2.8
Non-Hispanic	6.4% (478/7471)	19.4% (3140/16161)	3.0
**Rurality**			
Rural	5.8% (183/3129)	19.7% (1134/5749)	3.4
Urban	6.5% (317/4840)	18.4% (2484/13524)	2.8

## Discussion

We found email recruitment to be an effective, efficient, and equitable way to recruit VA patients to the LAMP study. The time to respond was consistently shorter for patients in the email group. The median response time of 1 day was shorter than the minimum estimated time to deliver postal mail. Response rates were higher for email recruitment overall and across individual subpopulations, including for older, non-White, Hispanic, rural, and female Veterans. Additionally, Black and multiracial patients had the largest ratio of email to postal response rates, highlighting the capabilities of using email to recruit populations often underrepresented in research.

The introductory postcards mailed to patients in the email recruitment group may have increased response rates by combining the benefits of digital and traditional recruitment methods, which has also been reported in other non-chronic-pain clinical trials [10, 11]. We heard from members of the study’s Veteran Engagement Panel, a diverse group of Veterans with chronic pain, that the introductory postcards lent credibility to the recruitment email, which would make the email less likely be disregarded, deleted, or marked as spam. Additionally, the recruitment email made it easy for patients to click on a link to access the study website, which would have required less effort than the patients who received the mail documents who had to manually enter the login information or use a QR code to access the study website. Overall, email recruitment combined with introductory postcards improved recruitment outcomes and reduced burden for both study staff and potential participants.

There were limitations to this study. We were not able to conduct a randomized controlled trial, which would have been the gold standard method to evaluate postal versus email recruitment. Due to the nature of the pragmatic clinical trial, different waves had different recruitment methods and were recruited at different times from different sites resulting in groups with different demographics. Response rates may have been affected by the different phases of the pandemic and other external factors at the time that each wave was recruited. Additionally, postal mail recruitment was only tested with patients from the Minneapolis VA site. Nevertheless, the multivariable analysis showed that response rates were greater for email recruitment after controlling for site (as well as age, gender, race, ethnicity, and rurality). Email-only recruitment (i.e., without postcards) was not tried with any of the waves, as this was not permitted by our Institutional Review Board (IRB). This study examined only VA patients, and the recruitment outcomes of email and mail recruitment might be different for non-VA populations. Other demographic factors that could impact recruitment, such as education status, socio-economic status, and household income were not available for analysis. Also, the study required interested participants to sign into a website, which may have been easier for those who received recruitment materials by email. We did not track the time required for email and postal recruitment and instead used an estimate. Finally, recruitment materials were sent during the COVID-19 pandemic, when people spent more time at home and might have been more likely to respond to recruitment materials.

## Conclusions

Email recruitment is an effective, efficient, and equitable way to recruit VA patients to large-scale, chronic pain clinical trials. Postal costs and personnel time were also much less for email recruitment. Future studies are needed to further explore how email recruitment affects groups who do not have regular access to email via computer or smartphone. As more VA studies consider using electronic recruitment and data collection, it will be important to ensure that all Veterans have access to resources that enable them to participate in VA research.

## Data Availability

The datasets used and/or analyzed during the current study are available from the corresponding author on reasonable request.

## Abbreviations

BPI: Brief Pain Inventory
COVID-19: Coronavirus disease 2019
DVAHCS: Durham VA Health Care System
EHR: Electronic health record
ICD-10-CM: International Classification of Diseases, Tenth Revision, Clinical Modification
IRB: Institutional Review Board
LAMP: Learning to Apply Mindfulness to Pain
MBI: Mindfulness-based intervention
MVAHCS: Minneapolis VA Health Care System
QR: Quick response
VA: Veterans Affairs
VAGLAHS: VA Greater Los Angeles Healthcare System
